# Comprehensive Knowledge about HIV/AIDS among Women of Reproductive Age in India

**DOI:** 10.3390/epidemiologia4040041

**Published:** 2023-11-16

**Authors:** Aritro Bhattacharyya, Ritankar Chakraborty, Tapasya Raj, Bijaya Kumar Padhi, Jagdish Khubchandani, Prakasini Satapathy, Sarvesh Rustagi, Vijay Kumar Chattu

**Affiliations:** 1 International Institute for Population Sciences, Mumbai 400088, India; aritro2506@gmail.com (A.B.); chakrabortyritankar@gmail.com (R.C.); tapasyarid@gmail.com (T.R.); 2 Postgraduate Institute of Medical Education and Research, Chandigarh 160012, India; bkpadhi@gmail.com; 3 Department of Public Health Sciences, New Mexico State University, Las Cruces, NM 88003, USA; 4 Center for Global Health Research, Saveetha Medical College and Hospital, Saveetha Institute of Medical and Technical Sciences, Saveetha University, Chennai 602105, India; prakasini.satapathy@gmail.com (P.S.); vkchattu@gmail.com (V.K.C.); 5 School of Pharmacy, Graphic Era Hill University, Dehradun 248007, India; 6 Department of Food Technology, Uttaranchal University, Dehradun 248007, India; sarveshrustagi@uumail.in; 7 Department of Occupation Science and Occupational Therapy-Temerty, Faculty of Medicine, University of Toronto, Toronto, ON P3C 1T6, Canada

**Keywords:** reproductive, sexual, HIV, AIDS, knowledge, India

## Abstract

HIV/AIDS has been a major threat to global public health, with India ranking third when it comes to the global burden of people living with HIV, especially women. It is imperative to assess the level of knowledge women have about transmission and prevention of this infection. This study sought to delineate the determinants of the comprehensive knowledge of HIV/AIDS among women in the reproductive age groups in India. Data from the fifth round of the National Family Health Survey conducted in India were analyzed. The sample included 95,541 women aged 15–49 years. Multilevel logistic regression was fitted with individual characteristics, household characteristics, and community characteristics to identify determinants of comprehensive knowledge on HIV/AIDS. Nearly a fourth (24.8%) of the women aged 15–49 in India who had ever heard of HIV had comprehensive knowledge of HIV/AIDS. Multilevel logistic regression showed that the likelihood of comprehensive knowledge of HIV/AIDS was higher among women aged 40–44 (AOR = 1.57) and 30–34 (AOR = 1.56). The likelihood of having comprehensive knowledge increased with the increase in the level of education. Women with secondary and higher levels of education were 1.9 times and 3.38 times more likely to have comprehensive knowledge, respectively, than those with no education. Household wealth, access to mass media, and having ever tested for HIV were also significant determinants of comprehensive knowledge of HIV/AIDS among women. The odds of having comprehensive knowledge about HIV/AIDS were higher for women with higher community wealth (AOR = 1.31), higher community education (AOR = 1.09), and higher community employment (AOR = 1.12). Factors at both the individual and community levels were shown to be indicators of comprehensive knowledge of HIV/AIDS. Policymakers and public health practitioners in India should come up with plans to close the information gaps about HIV/AIDS that exist among women and their demographic subgroups.

## 1. Introduction

As per the 2021 UNAIDS report, more than 38 million are living with HIV, with nearly 36.7 million of them being 15 years or older (the remaining 1.7 million are children). The majority (55%) of all global HIV infections are found among women. The overall death toll since the advent of the virus has reached nearly 40 million [1,2,3]. Geographically, the HIV pandemic has been harsh on middle- and low-income countries, especially in sub-Saharan Africa and South Asia [3,4,5,6,7]. Based on the World Health Organization and UNAIDS reports, some of the highest prevalence rates for HIV have been found in African countries such as Eswatini, Botswana, and Zimbabwe, among many others [1,2,3]. Considering the susceptible populations, the risk of developing HIV is higher among drug users, female sex workers, gay men, transgender women, and poorer individuals [8,9,10,11,12]. Along with the obvious trends in prevalence and susceptibility, socioeconomic disparities, health inequalities, gender inequality, abuse of power, and cultural hegemonies have further precluded comprehensive and coordinated global action to prevent HIV infections among women [13,14,15,16]. Gender inequality has deprived women of fundamental human rights, and the consequent disempowerment has led to a lack of economic, social, and sexual autonomy, burdening them with poorer health. Boosted by such inequalities, the risk of acquiring HIV infections for women has increased steadily and affects them disproportionately [4,5,6,7,8,9].

India, a country defined by its diversities and disparities, ranks third in terms of the global burden of people living with HIV [17]. The National AIDS Control Organization (NACO) of India had estimated a total of nearly 2.5 million people living with the infection as of 2021 [18]. The prevalence of HIV for Indian women in the 15–49 years age group is nearly 0.2%. More than 150,000 young people in the age group of 15–24 years have been living with HIV, while a total of 42,000 deaths have been recorded due to AIDS-related complications in India [17,18]. The National Family Health Survey also collects information on HIV/AIDS and, as per its latest (5th) round, nearly 87% of women and 94% of men have heard about HIV and its extreme consequences, which is a modest increase from the proportions reported in the previous rounds of the survey. For women, the knowledge of HIV has increased by 12%, and for men, it has increased by 5% since the previous survey cycle [19,20,21]. Despite this, continuous assessment and program planning are needed to increase awareness about HIV/AIDS.

Among women in middle-income and lower-income countries, where the societal inequalities are wider, tracking the proportion of young women with adequate HIV/AIDS knowledge can lead public health policies to be more inclusive, stringent, and focused, thereby providing greater opportunities for prevention [6,7,9,14]. In this regard, having ‘Comprehensive Knowledge of HIV/AIDS’ becomes essential as a part of the optimum reproductive and sexual health of individuals. Comprehensive knowledge of HIV encompasses knowing that consistent use of condoms during sexual intercourse and engaging exclusively with an uninfected sexual partner can prevent HIV infection, knowing that a healthy-looking person can have HIV, and rejecting two common misconceptions about the transmission and prevention of the virus. These two misconceptions are the ideas that HIV can be transmitted through mosquito bites and by sharing food with an already infected person [18,19,20,21]. As per the fourth round of the National Family Health Survey (NFHS 4, 2015–2016), about 21% of Indian women in the 15–49 years age group were found to have comprehensive knowledge of HIV. In India, HIV surveillance is one of the primary interventions in the national response to AIDS [19,20,21]. Still, a large proportion of people, mostly women, are living and dying because of HIV. There is an urgent need to conduct focused research on the determinants of comprehensive knowledge of HIV among Indian women in the context of socioeconomic, cultural, and demographic disparities. Thus, the purpose of this study was to assess the determinants of ‘Comprehensive Knowledge of HIV’ among women of reproductive age in India.

## 2. Methods

### 2.1. Data and Study Sample

We utilized data from the 5th round of the National Family Health Survey (NFHS-5) conducted in 2019–2020. The sample for NFHS-5 was designed to provide estimates of all key maternal and child health and nutrition indicators at the national and state levels, as well as estimates for selected indicators at the district level [19,22]. A sample size of 610,000 households across India was selected to produce reliable indicator estimates. A two-stage sampling procedure was adopted for both the rural and urban areas. In the rural areas, villages were selected as the primary sampling units (PSUs) in the first stage (selected with probability proportional to size), followed by a random selection of 22 households in each PSU in the second stage. In the urban areas, Census Enumeration Blocks (CEBs) were selected in the first stage and 22 households in each CEB were selected in the second stage. Complete mapping and household listing operations were performed on the first-stage units, from which the households were selected in the second stage in both the urban and rural areas. This study includes only those women who have ever heard of HIV/AIDS. A total of 108,785 women were asked if they had ever heard of AIDS, out of which 95,541 women replied with a ‘Yes’. After excluding women of reproductive age who had never heard of AIDS, a final sample of 95,541 women was used for analysis. The data are available at https://rchiips.org/nfhs/ (accessed 22 May 2023).

### 2.2. Measures

Comprehensive knowledge of HIV was our principal outcome of interest. According to the NFHS, comprehensive knowledge of HIV is the combined knowledge of different aspects pertinent to the spread and prevention of HIV. To ascertain the comprehensive knowledge of HIV, women in the age group of 15–49 years who declared themselves to be aware of HIV/AIDS were asked the following questions: Can using condoms during intercourse prevent the transmission of HIV? Can having one uninfected sexual partner prevent the transmission of HIV? Can HIV be transmitted through mosquito bites? Can HIV be transmitted by sharing food with an already infected person? Can a healthy-looking person have HIV? An individual was considered to have comprehensive knowledge of HIV/AIDS if they knew the correct response to all the five questions listed above. Correct response (coded as 1, compared to incorrect response coded as 0) for all the aforementioned questions meant that the woman had comprehensive knowledge of HIV. The outcome variable was recoded as a binary variable (yes = 1/no = 0) for the statistical analysis.

The independent variables were sociodemographic characteristics at the individual, household, and community levels. The individual-level variables included the age of the respondents, educational level, marital status, history of HIV testing (ever tested for HIV), current employment, and access to the internet. The ages of respondent women were grouped into 5-year age intervals as 15–19, 20–24, …, 45–49. Educational attainment for the respondents was categorized as ‘No Education’, ‘Primary’, ‘Secondary’, and ‘Higher’. Marital status was grouped as ‘Unmarried’, ‘Married’, and ‘Widowed, separated or divorced’. Ever tested for HIV, currently employed, and access to the internet, were dichotomously categorized into ‘Yes’ and ‘No’. The household-level variables included wealth index, caste, religion, and mass media exposure. The wealth index was taken as the proxy indicator for the household’s income. In NFHS, the wealth index was created using principal component analysis (PCA) of household wealth and assets and was divided into five quintiles—poorest, poorer, middle, richer, and richest. The household wealth variable was categorized as ‘Poor’, ‘Middle’, and ‘Rich’. The caste of the households was classified into ‘Scheduled Caste’, ‘Scheduled Tribe’, ‘Other Backward Classes’, and ‘Others’ (the caste system in India is a segmentation of society into groups whose membership is determined by birth). The religion of the households was categorized into ‘Hindu’, ‘Muslim’, ‘Christian’, and ‘Others’. Exposure to mass media was defined as the combined exposure to newspaper, radio, and television (at least once a week), estimated at a household level and dichotomously categorized as ‘Having exposure’ and ‘Not having exposure’.

Community-level variables included the place of residence, community wealth, community education, and community employment. Place of residence was dichotomously classified into ‘Urban’ and ‘Rural’. Community wealth, education, and employment were categorized as ‘Low’ and ‘High’. The community-level variables were estimated by aggregating the individual- or household-level characteristics to the primary sampling unit (PSU) level. The community wealth variable was dichotomized into ‘High’ and ‘Low’, where high was for those PSUs whose average wealth index (WI) was greater than the national average of WI, and low was for those PSUs whose average WI was less than the national average of WI. Community education and community employment were also generated and recoded similarly.

### 2.3. Statistical Analysis

The study used bivariate analysis to examine the relationship of comprehensive knowledge of HIV/AIDS with demographic and socio-economic correlates. A chi-square test was performed to test this relationship. To understand how comprehensive knowledge of HIV/AIDS is associated with socioeconomic and demographic correlates, we fitted a multilevel logistic regression with individual characteristics as level 1, household characteristics as level 2, and community characteristics as level 3. The random effects of household and community levels were estimated using the *melogit* command in STATA (version 16). Four models were fitted for the outcome variable. The first model, called the empty/null model (Model 0), did not include any exposure variables. It focused on decomposing the total variance at the household and community levels. The intra-class correlation coefficient (ICC), which is a useful measure to determine the extent of cluster variation in the dependent variable, was calculated. The second model (Model 1) controlled for the individual-level covariates, the third (Model 2) model was composed of the individual- as well as household-level covariates, while the fourth model (Model 3) included all the socio-demographic covariates at the individual, household, and community levels. Odds ratios with 95% confidence intervals (CIs) have been reported with *p*-values determining the level of significance. The mathematical description of the final model (two levels) is given below:

$$logit\;\left(\pi_{ijk}\right)=\log(\frac{\pi_{ijk}}{1-\pi_{ijk}})=\;\beta_{0jk}+\;\beta_{1}x_{1ijk}+\;\beta_{2}x_{2ijk}+\cdots +\;\beta_{n}x_{nijk}$$

Here, $\pi_{ijkl\;}=p\left(y_{ijk}=1\right)$ is the probability that a woman (*i*), in the household (*j*), from the PSU (*k*), had comprehensive knowledge of HIV/AIDS. Here, *y_ijk_* is equal to 1 if the woman had comprehensive knowledge and is equal to 0 if she had no knowledge. $\beta_{0jk}$ is the random intercept term at the household and PSU levels. The advantage of this method is that one error term is generated at each level, which allows us to isolate the residual variances at each level.

## 3. Results

### 3.1. Comprehensive Knowledge about HIV/AIDS and Sociodemographic Characteristics

Table 1 shows the association of comprehensive knowledge of HIV/AIDS among women aged 15–49 years with different socio-economic and demographic characteristics. Nearly a fourth (24.8%) of the women aged 15–49 in India who had ever heard of HIV had comprehensive knowledge of HIV/AIDS. Comprehensive knowledge of HIV/AIDS was higher among women in the age groups of 20–24 (25%), 25–29 (27%), and 30–34 (28%) years as compared to women of the other age groups. Marital status was significantly associated (*p* < 0.001) with having comprehensive knowledge, with unmarried women having slightly better knowledge than married women. Almost 38% and 25% of the women with higher and secondary education, respectively, had comprehensive knowledge of HIV/AIDS, as compared to only 19% and 15% for women with primary and no education, respectively. Women who had ever been tested for HIV and had access to mass media had a higher likelihood of having comprehensive knowledge of HIV/AIDS compared to those who had never been tested and had no access to mass media (*p* < 0.001). Nearly a third (31%) of the women in rich households had comprehensive knowledge, as compared to 23% and 17% for the women in middle and poor households, respectively. Place of residence was also found to have a significant association with comprehensive knowledge of HIV/AIDS among women (higher among urban women than rural women). For community-level variables, women belonging to communities with levels of education, wealth, and employment higher than the respective national averages had a higher likelihood of having comprehensive knowledge of HIV/AIDS compared to their counterparts.

### 3.2. Individual-, Household-, and Community-Level Predictors of HIV/AIDS Comprehensive Knowledge

Table 2 demonstrates the results from the full model of the multilevel regression which included all the individual-, household-, and community-level variables. Our analysis found that the age of the women, marital status, education level, history of testing for HIV, household wealth, mass media exposure, community wealth, community education, and community employment had a significant association with whether they had comprehensive knowledge of HIV/AIDS.

The likelihood of having comprehensive knowledge of HIV/AIDS was highest among women in the age groups of 40–44 years (AOR = 1.57, 95% CI = 1.39, 1.76) and 30–34 years (AOR = 1.56, 95% CI = 1.40–1.74). Married women (AOR = 0.85, 95% CI = 0.78–0.92) and divorced/widowed/separated women (AOR = 0.78, 95% CI = 0.68–0.90) were less likely to have comprehensive knowledge of HIV/AIDS when compared to unmarried women. Women who had attained a primary level of education were 1.28 times more likely to have comprehensive HIV/AIDS knowledge compared to women who did not attain any education (AOR = 1.28, 95% CI = 1.17–1.40). The likelihood of having comprehensive knowledge increased with the increase in the level of education. Women with a secondary level of education were 1.9 times more likely (AOR = 1.92, 95% CI = 1.77–2.07) and women with a higher level of education were 3.38 times more likely (AOR = 3.38, 95% CI = 3.06–3.75) to have comprehensive knowledge of HIV/AIDS.

Household wealth was found to be a significant determinant of having comprehensive knowledge of HIV/AIDS. The likelihood of having knowledge increased significantly as household wealth increased, with women belonging to rich and middle households having 1.28 times and 1.21 times higher odds of being knowledgeable than women from poorer households. Women who had ever tested for HIV had significantly higher odds of having comprehensive knowledge about HIV/AIDS (AOR = 1.62, 95% CI = 1.52–1.71). Women who had mass media exposure were more likely to have comprehensive knowledge about HIV/AIDS (AOR = 1.19, 95% CI = 1.11–1.28). Women residing in communities with a level of community wealth, level of community education, and level of community employment higher than the respective national averages were more likely to have comprehensive knowledge of HIV than their counterparts. The odds of having comprehensive knowledge of HIV/AIDS were higher for women with higher community wealth (AOR = 1.31, 95% CI = 1.19–1.45), community education (AOR = 1.09, 95% CI = 1.00–1.20), and community employment (AOR = 1.12, 95% CI = 1.03–1.21).

The variation in comprehensive knowledge of HIV/AIDS among women across the individual, household, and community levels is shown in Table 3. A model applied without any covariates (null model) showed a significant amount of variation in comprehensive knowledge of HIV/AIDS among women across the household and community levels. The intra-class correlation coefficient (ICC) values showed that 55% and 29% of the total variation in comprehensive knowledge of HIV/AIDS among women was attributable to differences across households and communities, respectively. Adding all the individual-, household-, and community-level predictors to the null model reduced the value of ICC. ICC values from the full model indicated that 53% and 26% of the total variation in comprehensive knowledge of HIV/AIDS among women was attributable to differences across the household and community levels, respectively.

## 4. Discussion

There are several salient features of this national assessment in India. First, education is a key factor in women having comprehensive knowledge of HIV/AIDS in the reproductive age group. Women with higher levels of education were found to have better knowledge than those with little to no education. Second, having ever tested for HIV and having access to mass media were major and significant determinants of comprehensive knowledge of HIV/AIDS among women. Third, there is a rural–urban gap in comprehensive knowledge, with women in urban areas better equipped with resources to gain knowledge about HIV/AIDS than their rural counterparts. Lastly, community education, community wealth, and community employment have a significant role to play in women having comprehensive knowledge about HIV/AIDS. The higher the level of employment, education, and wealth of a community, the higher the chances of women having comprehensive knowledge about HIV/AIDS.

The odds of having comprehensive knowledge of HIV/AIDS were higher among women with higher levels of education than those with no or primary education. This finding is in line with other studies conducted in several African countries and parts of Asia [23,24,25,26,27,28,29,30]. Even a study conducted in India exclusively with a slum population found that people who pursued education for a greater number of years had a significantly higher likelihood of having comprehensive knowledge of HIV/AIDS compared to those with fewer years or no education [31]. This can be attributed to the fact that educated women are more aware of their health than those with little to no education. Individuals may get information on HIV/AIDS from school-based interventions. Education on reproductive or sexual health in academic institutions is significantly associated with greater knowledge about HIV/AIDS [32].

Our study indicates that women who had ever been tested for HIV had a higher likelihood of having comprehensive knowledge of HIV/AIDS. This is consistent with other studies worldwide that show that women who had been tested for HIV were able to gain knowledge about HIV prevention by using community healthcare or HIV-related counseling services [33,34]. Additional knowledge about HIV/AIDS can be provided to women when they have access to HIV counseling and testing services. Some studies have highlighted that knowing where to be tested for HIV has a significant and positive association with comprehensive knowledge of HIV/AIDS among women [33,34,35]. Providing pre-test information and post-test counseling on the key principles of HIV testing and counseling creates an opportunity for individuals to gather information regarding HIV/AIDS and enables them to remove preconceived notions [33,34,35,36]. Therefore, the widespread presence of HIV counseling and testing facilities can help increase the comprehensive knowledge about HIV/AIDS among women in India.

Women with access to mass media were more likely to have comprehensive knowledge of HIV/AIDS than those who had no exposure to mass media. Although conducted in different populations (e.g., youth and males), our findings are consistent with a few studies from Sub-Saharan African countries and South Asian countries [15,25,26,27,28,29,30,37,38,39]. Television, newspapers/magazines, and radio are important media tools to disseminate critical information to the general population. Social networks are also important pathways to promote knowledge about HIV/AIDS, particularly among those in the middle age groups. Similarly, the rural–urban gap in comprehensive knowledge of HIV/AIDS found in this study could be because people residing in urban areas have greater access to health information, media outlets, and healthcare facilities. In addition, people in urban areas have greater exposure to campaigns and programs related to HIV/AIDS, and HIV/AIDS prevention and control interventions such as testing and counseling campaigns and training sessions [25,27,29,31,34,37,39]. Therefore, greater awareness efforts about HIV/AIDS can be directed toward rural women in developing countries.

The most insightful and novel finding from our study is that community-level variables such as higher community education, community wealth, and community employment had a statistically significant association with comprehensive knowledge of HIV/AIDS among women. Residing in communities with higher wealth where most people are educated and employed increases the likelihood of women knowing about HIV/AIDS. Wealthier families have better access to mass media outlets and social networking, which are major pathways to procuring information regarding HIV/AIDS. Educated families are more aware of the ways of preventing HIV/AIDS and have better access to HIV testing and counseling facilities concentrated in such areas. Better employment ensures improved living standards along with a preference for attaining higher education, and subsequently boosted access to information, communication, and healthcare facilities, in contrast to unemployed people [24,27,34].

India has progressed tremendously in its efforts to eradicate HIV/AIDS, which poses major threats to the large population of the country in the reproductive age group [17,18,19,20,21]. The HIV prevalence has already reduced quite significantly over the years, but sustained efforts, which are still needed to raise awareness among people about its occurrence and spread, are very significant in protecting people from the epidemic. The National AIDS Control Programme (NACP) lays maximum emphasis on the widespread reach of information, education, and communication about HIV/AIDS prevention [18,20]. Despite improvements over the last few years in the awareness of HIV/AIDS among women, the percentage of women with comprehensive knowledge of HIV/AIDS remains alarmingly low, given the fact that women are at a higher risk of having HIV/AIDS. Community participation holds the key to increasing knowledge about HIV/AIDS among women. Discussions about the spread and prevention of HIV/AIDS in public forums, during cultural and religious events, or via public service announcements and media campaigns can help increase the knowledge of HIV/AIDS among women in India.

The results of this study should be interpreted considering a few potential limitations. NFHS provides cross-sectional data, which does not allow causal interpretation between variables. Moreover, the use of a secondary dataset limits us to the use of only those variables that are available for analysis. The study data are also subject to recall bias and social desirability from the respondents. HIV/AIDS is a sensitive topic and is still considered taboo in some parts of the country. When seeking out data on sensitive topics, the resultant data quality is often compromised because sensitive topics are more prone to errors related to non-response and measurement. All the traditional limitations of a cross-sectional design apply to our findings (e.g., threats to validity and reliability). Despite these limitations, the study has many strengths. The use of multilevel modeling techniques gave us the fixed effects of the individual-, household-, and community-level variables. The multilevel analysis also helped us understand the variation in comprehensive knowledge of HIV/AIDS among women attributable to different levels. Finally, the use of a national database provides highly generalizable findings for stakeholders.

## 5. Conclusions

This study sought to assess the proportion and determinants of comprehensive knowledge of HIV/AIDS among women aged 15–49 years in India. Findings from this study indicate that several individual-, household-, and community-level factors can influence comprehensive knowledge of HIV/AIDS among women. From a policy perspective, we recommend that government agencies and other concerned authorities in India should not limit their focus to individual characteristics but also consider the household- and community-level characteristics during policy making and program planning for HIV awareness. Strategies should be designed in a way to address the knowledge gaps among women and should target the underprivileged population sub-groups. Mass media campaigns and community-based educational interventions with a focus on rural, less educated, and unemployed women should be implemented in a sustained and culturally tailored manner. Additionally, enhanced facilities for testing and counseling of HIV/AIDS specifically for marginalized women should help in improving comprehensive knowledge of HIV/AIDS among women of reproductive age in India.

## Figures and Tables

**Table 1:** Sociodemographic characteristics of the study population.

Characteristics	N	HIV/AIDS Comprehensive Knowledge		*p*-Value
		Yes (%)	No (%)	
**Total**	95,541	24.79	75.21	
**Age Groups (years)**				<0.001
15–19	15,437	21.71	78.29	
20–24	15,983	25.38	74.62	
25–29	16,012	26.99	73.01	
30–34	13,756	27.67	72.33	
35–39	12,998	24.42	75.58	
40–44	10,455	24.44	75.56	
45–49	10,900	22.18	77.82	
**Marital Status**				<0.001
Unmarried	24,008	25.37	74.63	
Married	67,485	24.80	75.20	
Divorced/Widowed/Separated	4048	21.27	78.73	
**Education Level**				<0.001
No education	19,348	15.33	84.67	
Primary	10,686	18.76	81.24	
Secondary	50,312	25.14	74.86	
Higher	15,195	37.98	62.02	
**Currently Employed**				0.001
No	70,566	24.35	75.65	
Yes	24,975	26.06	73.94	
**Internet Use**				<0.001
No	60,856	20.18	79.82	
Yes	34,685	32.73	67.27	
**Tested for HIV**				<0.001
No	73,648	21.95	78.05	
Yes	21,893	33.58	66.42	
**Household Wealth Quintile**				<0.001
Poor	38,073	17.48	82.52	
Middle	20,513	23.46	76.54	
Rich	36,955	31.23	68.77	
**Caste**				<0.001
SC	17,803	22.53	77.47	
ST	17,892	24.04	75.96	
OBC	36,995	23.87	76.13	
Others	18,357	29.58	70.42	
**Religion**				<0.001
Hindu	71,692	25.14	74.86	
Muslim	11,666	20.23	79.77	
Christian	7375	31.69	68.31	
Others	4808	29.81	70.19	
**Mass Media Exposure**				<0.001
No	19,595	15.64	84.36	
Yes	75,946	26.94	73.05	
**Place of Residence**				<0.001
Urban	25,210	30.78	69.22	
Rural	70,331	21.63	78.37	
**Community Wealth**				<0.001
Low	46,481	18.4	81.6	
High	49,060	29.45	70.55	
**Community Education**				<0.001
Low	52,814	19.8	80.2	
High	42,727	30.36	69.64	
**Community Employment**				<0.001
Low	53,567	23.16	76.84	
High	41,974	26.94	73.06	

**Table 2:** Individual-, household-, and community-level predictors of comprehensive knowledge on HIV/AIDS.

Predictors	Model 1 AOR (95% CI)	Model 2 AOR (95% CI)	Model 3 AOR (95% CI)
**Age (years)**			
15–19	Ref	Ref	Ref
20–24	1.09 (1–1.18) *	1.10 (1.00–1.20) *	1.10 (1.01–1.20) *
25–29	1.33 (1.21–1.47) **	1.31 (1.18–1.45) **	1.30 (1.18–1.44) **
30–34	1.68 (1.51–1.86) **	1.59 (1.42–1.77) **	1.56 (1.40–1.74) **
35–39	1.66 (1.49–1.85) **	1.56 (1.39–1.74) **	1.52 (1.36–1.70) **
40–44	1.73 (1.55–1.94) **	1.61 (1.43–1.81) **	1.57 (1.40–1.76) **
45–49	1.74 (1.56–1.96) **	1.58 (1.40–1.78) **	1.54 (1.36–1.73) **
**Marital Status**			
Unmarried	Ref	Ref	Ref
Married	0.82 (0.76–0.89) **	0.84 (0.77–0.91) **	0.85 (0.78–0.92) **
Divorced/Widowed/Separated	0.76 (0.66–0.86) **	0.78 (0.68–0.90) **	0.79 (0.69–0.90) **
**Education Level**			
No education	Ref	Ref	Ref
Primary	1.37 (1.26–1.5) **	1.29 (1.18–1.41) **	1.28 (1.17–1.40) **
Secondary	2.22 (2.07–2.39) **	1.94 (1.79–2.09) **	1.92 (1.77–2.07) **
Higher	4.16 (3.78–4.58) **	3.45 (3.11–3.81) **	3.39 (3.06–3.75) **
**Currently Employed**			
No	Ref	Ref	Ref
Yes	1.06 (1.01–1.12) *	1.07 (1.01–1.13) *	1.05 (0.99–1.11)
**Internet Use**			
No	Ref	Ref	Ref
Yes	1.61 (1.52–1.70) **	1.49 (1.41–1.58) **	1.48 (1.39–1.57) **
**Tested for HIV**			
No	Ref	Ref	Ref
Yes	1.68 (1.59–1.78) **	1.65 (1.56–1.75) **	1.62 (1.52–1.71) **
**Household Wealth Quintile**			
Poor (Ref)		1	1
Middle		1.29 (1.20–1.39) **	1.21 (1.12–1.30) **
Rich		1.46 (1.36–1.58) **	1.28 (1.18–1.39) **
Caste			
SC (Ref)		1	1
ST		1.25 (1.13–1.38) **	1.30 (1.18–1.44) **
OBC		1.07 (0.99–1.15)	1.07 (1.00–1.16)
Others		1.16 (1.07–1.27) **	1.17 (1.07–1.27) **
**Religion**			
Hindu (Ref)		1	1
Muslim		0.80 (0.72–0.89) **	0.80 (0.72–0.89) **
Christian		1.15 (1.01–1.31) *	1.14 (1.00–1.29)
Others		0.88 (0.76–1.01)	0.86 (0.75–0.99) *
**Mass Media Exposure**			
No (Ref)		1	1
Yes		1.22 (1.14–1.31) **	1.20 (1.11–1.28) **
**Place of Residence**			
Urban			1
Rural			0.94 (0.85–1.04)
**Community Wealth**			
Low (Ref)			1
High			1.31 (1.19–1.45) **
**Community Education**			
Low (Ref)			1
High			1.10 (1.00–1.20) *
**Community Employment**			
Low (Ref)			1
High			1.12 (1.03–1.21) **

* Indicates *p* < 0.01 and ** indicates *p* < 0.001. AOR (95% CI) indicates adjusted odds ratios for the outcome with 95% confidence intervals. Ref indicates the reference group for the predictor variable.

**Table 3:** Variance estimation for household- and community-level predictors of comprehensive knowledge about HIV/AIDS.

	Null Model	Model 1	Model 2	Model 3
Community level variance	2.124 (0.069)	1.925 (0.065)	1.862 (0.065)	1.855 (0.064)
HH level variance	1.845 (0.090)	1.864 (0.092)	1.878 (0.095)	1.871 (0.095)
PSU level ICC	0.29	0.27	0.26	0.26
HH level ICC	0.55	0.54	0.53	0.53

ICC indicates intra-class coefficients. PSU—primary sampling unit indicating community level and HH indicates household level.

## Data Availability

The data are available at https://rchiips.org/nfhs/ (accessed on 22 May 2023).
